# *OsMFT1* increases spikelets per panicle and delays heading date in rice by suppressing *Ehd1*, *FZP* and *SEPALLATA*-like genes

**DOI:** 10.1093/jxb/ery232

**Published:** 2018-06-21

**Authors:** Song Song, Guanfeng Wang, Yong Hu, Haiyang Liu, Xufeng Bai, Rui Qin, Yongzhong Xing

**Affiliations:** 1 National Key Laboratory of Crop Genetic Improvement, Huazhong Agricultural University, Wuhan, China; 2 Key Laboratory of State Ethnic Affairs Commission for Biological Technology, College of Life Sciences, South-Central University for Nationalities, Wuhan, China

**Keywords:** Branch meristem, *Ghd7*, heading date, *OsLFL1*, *OsMFT1*, panicle architecture, spikelet meristem

## Abstract

Heading date and panicle architecture are important agronomic traits in rice. Here, we identified a gene *MOTHER OF FT AND TFL1* (*OsMFT1*) that regulates rice heading and panicle architecture. Overexpressing *OsMFT1* delayed heading date by over 7 d and greatly increased spikelets per panicle and the number of branches. In contrast, *OsMFT1* knockout mutants had an advanced heading date and reduced spikelets per panicle. Overexpression of *OsMFT1* significantly suppressed *Ehd1* expression, and *Ghd7* up-regulated *OsMFT1* expression. Double mutants showed that *OsMFT1* acted downstream of *Ghd7*. In addition, transcription factor OsLFL1 was verified to directly bind to the promoter of *OsMFT1* via an RY motif and activate the expression of *OsMFT1 in vivo* and *in vitro*. RNA-seq and RNA *in situ* hybridization analysis confirmed that *OsMFT1* repressed expression of *FZP* and five *SEPALLATA*-like genes, indicating that the transition from branch meristem to spikelet meristem was delayed and thus more panicle branches were produced. Therefore, *OsMFT1* is a suppressor of flowering acting downstream of *Ghd7* and upstream of *Ehd1*, and a positive regulator of panicle architecture.

## Teaser

A new rice gene, *OsMFT1*, was identified that both regulates rice heading date and panicle architecture and is different from its homologs in other species.

## Introduction

Heading date (flowering time) in rice (*Oryza sativa*) is crucial for plants to adapt to the growing environment and for the improvement of yield potential. It is determined by the interaction of endogenous signals and environmental factors. Florigen is a key endogenous signal that is synthesized in the leaves and moves to the shoot apex to induce flowering. Many environmental factors could induce or suppress florigen expression. Photoperiod (day length) is the most important environmental factor affecting flowering time (Song *et al.*, 2015). Rice is a typical short-day (SD) plant, one whose flowering is promoted by short daylength. Many genes have been identified as involved in the photoperiod-mediated flowering pathway. *Heading date 3a* (*Hd3a*) and *RICE FLOWERING LOCUS T1* (*RFT1*), which are homologous to *Arabidopsis thaliana FLOWERING LOCUS T* (*FT*), are florigen genes of rice (Kojima *et al.*, 2002; Tamaki *et al.*, 2007; Komiya *et al.*, 2008; Komiya *et al.*, 2009). In rice, there are two pathways determining floral induction. One is the *Heading date 1* (*Hd1*)*–Hd3a* pathway, which is conserved with the Arabidopsis *CONSTANS* (*CO*)–*FT* pathway. The other is a unique rice pathway, the *Ghd7–Ehd1–Hd3a/RFT1* pathway (Song *et al.*, 2015). *Hd1*, homologous to Arabidopsis *CO*, promotes *Hd3a* expression under SD conditions and suppresses *Hd3a* under long-day (LD) conditions (Yano *et al.*, 2000). *Early heading date 1* (*Ehd1*) activates *Hd3a* and *RFT1* expression independent of *Hd1* in both LD and SD conditions (Doi *et al.*, 2004). *Grain number*, *plant height*, *and heading date 7* (*Ghd7*) suppresses *Ehd1* expression. *Ghd7* expression is sensitive to photoperiod. Under LD conditions, *Ghd7* expression is induced and thus *Ehd1* and *Hd3a* expression is suppressed. Under SD conditions, *Ghd7* has low expression and the suppression of *Ehd1* is relieved, which allows *Ehd1* to induce *Hd3a* expression (Xue *et al.*, 2008). In rice, a number of flowering genes are found to function by directly or indirectly regulating *Ehd1*, and thus *Ehd1* acts as a floral integrator (Song *et al.*, 2015). A recent discovery showed that Ghd7 interacts with Hd1 to delay heading, which indicates these two pathways are not independent in regulating heading date (Nemoto *et al.*, 2016; Zhang *et al.*, 2017).

The rice inflorescence has a branch structure and is usually referred to as a panicle. A rice panicle consists of a main stem, often referred to as the rachis, several primary branches, one or more secondary branches on the primary branch, and occasionally tertiary branches on the secondary branch (Xing and Zhang, 2010). After the transition from the vegetative phase to the reproductive phase, the shoot apical meristem is converted into the inflorescence meristem, and then the inflorescence meristem produces branch meristems. Each branch meristem can continue to produce new branch meristems or transform into a spikelet meristem, and then the spikelet meristem is converted to a floral meristem. In rice, each spikelet meristem generates one flower. Therefore, spikelet meristem identity determines the termination of branch meristem activity. Currently, many genes have been identified as being responsible for the initiation, maintenance, and activity of these meristems (Tanaka *et al.*, 2013; Zhang and Yuan, 2014). A major yield quantitative trait locus (QTL), *GRAIN NUMBER 1a* (*GN1a*), encodes a cytokinin oxidase, OsCKX2. High levels of cytokinin caused by loss-of-function of *OsCKX2* increase branch meristem activity, which is responsible for the increased branch and spikelet number (Ashikari *et al.*, 2005). Rice *FRIZZY PANICLE* (*FZP*) regulates the transition of branch meristems to spikelet meristems and has a crucial role in establishing the spikelet meristem identity. *fzp* mutants produce branches but fail to form normal spikelets, and spikelets are replaced by branches in mutants carrying severe *fzp* alleles (Chujo *et al.*, 2003; Komatsu *et al.*, 2003; Bai *et al.*, 2016). Another rice yield gene, *TAWAWA1* (*TAW1*), also regulates spikelet number through suppression of the transition from branch meristems to spikelet meristems. *TAW1* promotes branch meristem activity and suppresses the phase change to spikelet meristem identity through positively regulating the *SVP* family MADS-box genes, leading to prolonged branch formation (Yoshida *et al.*, 2013). Thus, promotion of inflorescence meristem or branch meristem activity and appropriate delay of spikelet meristem identity formation could help to increase spikelet number.

Rice *MOTHER OF FT AND TFL1* (*MFT*) belongs to the family of phosphatidylethanolamine-binding proteins (PEBPs). The PEBP family is a family of evolutionarily conserved genes widely present in eukaryotes (Karlgren *et al.*, 2011). In higher plants, the PEBP gene family consists of three main homologous subfamilies, *FT*-like, *TERMINAL FLOWER1* (*TFL1*)-like and *MFT*-like genes (Chardon and Damerval, 2005). As the name suggests, the *MFT*-like subfamily is a homolog of *FT* and *TFL1* and is thought of as the evolutionary ancestor to them (Hedman *et al.*, 2009). In Arabidopsis, there are six PEBP family genes: two *FT*-like genes (*FT* and *TSF*), three *TFL*-like genes (*TFL1*, *BFT*, and *ATC*) and one *MFT*-like gene (*MFT*) (Danilevskaya *et al.*, 2008). Both FT and TFL1 are key regulators of floral transition but have antagonistic roles. FT has been shown to be florigen and induces flowering while TFL1 has been identified as a flowering suppressor (Alvarez *et al.*, 1992; Kardailsky *et al.*, 1999; Hanzawa *et al.*, 2005). In addition to repressing flowering, *TFL1* plays a crucial role in determining inflorescence architecture. In Arabidopsis, a main shoot apical meristem produces either indeterminate flowers or indeterminate lateral axes after floral transition. *TFL1* prevents the meristems from assuming the floral identity and accounts for indeterminate growth of the inflorescence shoot. Thus, 35S::*TFL1* transgenic plants exhibited an extended vegetative phase and branched inflorescence while loss-of-function of *TFL1* produced terminal flowers at the shoot apex (Alvarez *et al.*, 1992; Benlloch *et al.*, 2007; Liu *et al.*, 2013). *ARABIDOPSIS THALIANA CENTRORADIALIS* (*ATC*) and *BROTHER OF FT AND TFL1* (*BFT*) inhibit flowering similarly to *TFL1* (Huang *et al.*, 2012; Yoo *et al.*, 2010), and *TSF* promotes flowering similarly to *FT* (Yamaguchi *et al.*, 2005). As a gene homologous to both *FT* and *TFL1*, *MFT* seems to have no major effect on flowering. Overexpression of *MFT* led to slightly early flowering while loss of *MFT* function did not exhibit an obvious phenotype in flowering (Yoo *et al.*, 2004). Later studies showed *MFT* is involved in the regulation of seed germination via ABA and GA signaling pathways. Loss-of-function of *MFT* led to hypersensitivity to ABA in seed germination. *MFT* is directly bound by *ABA-INSENSITIVE 3* (*ABI3*) and *ABI5* on the promoter. *MFT* is suppressed and promoted by *ABI3* and *ABI5*, respectively. In addition, DELLA proteins, the major repressors of GA signaling, could directly bind to the *MFT* promoter and promote its expression. On the other hand, *MFT* exerts a negative feedback regulation of ABA signaling by directly repressing *ABI5* (Xi *et al.*, 2010). Besides Arabidopsis, there are several *MFT* homologs reported to regulate seed germination in other species. In wheat, *TaMFT* is a repressor of seed germination and co-localizes with a seed dormancy QTL (Nakamura *et al.*, 2011). Through ectopic overexpression in Arabidopsis, a Soyben homolog of *MFT* (*GmMFT*) negatively regulates seed germination, and strawberry homolog of *MFT* (*FvMFT*) regulates germination via participating in GA and ABA signaling (Li *et al.*, 2014; Hu *et al.*, 2016).

In rice, 19 PEBP genes were identified based on genome wide analysis, of which there were 13 *FT*-like genes, four *TFL*-like genes, and two *MFT*-like genes (Chardon and Damerval, 2005; Danilevskaya *et al.*, 2008). Among them, the most well-studied homolog of *FT* is *Hd3a*. Ha3a is a mobile flowering signal that moves from leaf to shoot apical meristem where it interacts with 14-3-3 protein and OsFD1 to form a florigen activation complex, which is essential for the activation of the inflorescence meristem identity gene (Tamaki *et al.*, 2007; Taoka *et al.*, 2011). The four *TFL1* homologs in rice are named *RCN1*, *RCN2*, *RCN3*, and *RCN4*. Overexpression of *RCN1*, *RCN2*, and *RCN3* exhibited branched dense panicle architecture and delayed heading date, while knocking down of all RCNs produced reduced branches and small panicles (Nakagawa *et al.*, 2002; Zhang *et al.*, 2005; Liu *et al.*, 2013). So far, two *MFT* homologs in rice, *OsMFT1* and *OsMFT2*, have not been identified yet. A previous study proposed that *OsMFT1* was positively regulated by *Ghd7* in the flowering pathway through an expression QTL (eQTL)-guided function-related co-expression analysis (Wang *et al.*, 2014). It is very likely that *OsMFT1* regulates heading date and panicle architecture. Here, we confirmed that *OsMFT1* acts downstream of *Ghd7* and elucidated its mechanism in controlling heading and panicle architecture by identification of its upstream and downstream genes.

## Materials and methods

### Plant material and growth condition

The japonica rice variety Zhonghua 11 (ZH11) was used as the wild type and recipient for genetic transformation. *Ghd7*-related material, including NIL(mh7), NIL(zs7), OX-*Ghd7*^ZH11^, and Ami-*Ghd7*, were from previous studies (Xue *et al.*, 2008; Weng *et al.*, 2014); the *ghd7* mutant had an SNP mutation resulting in a premature stop codon in the ZH11 background. For measurement of the agronomic traits, rice plants grown at Wuhan were under natural LD conditions, whereas plants grown at Hainan were under SD conditions. Germinated seeds were sown in the seed beds and 1-month-old seedlings were transplanted to the fields with 10 plants in a row. The heading date was the day when the first panicle of the plant emerged. Plants in the middle of each row were harvested individually and used to score the traits of spikelets per panicle, number of primary branches, and number of secondary branches.

### Vector construction and genetic transformation

To generate the overexpression vector, coding sequences of *OsMFT1* were isolated from ZH11 leaf cDNA and cloned into T-vector (Promega), then digested with *Kpn*I and *Xba*I and cloned into the *Kpn*I–*Xba*I sites of pCAMBIA1301S. For generating CRISPR mutants, a specific single guide RNA (sgRNA) targeting *OsMFT1* was designed and assembled into the vector pCXUN-CAS9 (sgRNA was driven by the U3 promoter). The constructs were introduced into ZH11 callus by Agrobacterium-mediated transformation.

### RNA extraction and qRT-PCR analysis

Samples of leaves, young panicles, and other tissues were frozen in liquid nitrogen immediately after being collected from the plants. Total RNA was extracted using Trizol reagent (TransGen Biotech, Beijing). Then 3 μg of total RNA was digested by DNase I and reverse transcribed by Superscript III reverse transcriptase (Invitrogen, USA) to obtain the first-strand cDNA according to the manufacturer’s protocol. Real-time PCR was performed in a 96-well plate in an ABI Prism 7500 real-time PCR system (Applied Biosystems, USA) using SYBR Premix ExTaq reagent (TaKaRa, Dalian). The relative expression levels were calculated according to the method proposed previously (Livak and Schmittgen, 2001), with the rice ubiquitin gene serving as an internal control. Primers used for real-time PCR are listed in Supplementary Table S1 at *JXB* online.

### Subcellular localization

To confirm the subcellular localization of *OsMFT1*, the coding sequence of *OsMFT1* was amplified and inserted into the pM999 vector driven by the CaMV 35S promoter. The fusion construct 35S::*OsMFT1*::YFP was co-transformed into rice protoplasts with 35S::*GHD7*::CFP, which was used as a nuclear marker. The construct 35S::YFP was used as a control. Rice protoplasts transformation was conducted as previously described (Xie and Yang, 2013). After transformation into rice protoplasts and incubation in the dark for 12–16 h, the fluorescence was observed by confocal microscopy (Leica Microsystems).

### Yeast one-hybrid assay

AD-*OsLFL1* was constructed by inserting the coding sequence of *OsLFL1* into the vector pB42AD (Clontech, USA). The promoter fragments of *OsMFT1* were cloned into the vector pLacZi2μ to construct pro::LacZ. The yeast one-hybrid assay was performed as previously described (Tang *et al.*, 2012). Briefly, the AD-*OsLFL1* and pro::LacZ were co-transformed into yeast strain EGY48 and spread on the selective medium SD/−Trp/−Ura (Clontech). The grown transformants were transferred to SD/−Trp/−Ura medium containing raffinose, galactose, and X-gal (Sigma-Aldrich, USA) for developing the blue color.

### Electrophoretic mobility shift assay

To get the OsLFL1 protein, the coding sequence of *OsLFL1* was amplified and cloned into the expression vector pSPUTK (Smaczniak *et al.*, 2012). Proteins were synthesized using the TNT SP6 High-Yield Wheat Germ Protein Expression System (Promega). The oligonucleotides were synthesized and labeled with 5′-biotin by the Shanghai Sangon Company. Double-stranded oligonucleotides were generated by mixing equal amounts of the complementary single-stranded oligonucleotides and heating for 2 min at 95 °C, then cooling down to 25 °C. Biotin-labeled probes were incubated with the OsLFL1 protein in the binding buffer [10 mM Tris (pH 7.5), 50 mM KCl, 1 mM EDTA, 5 mM MgCl_2_, 1 mM DTT, 50 ng μl^−1^ Poly (dI–dC), 2.5% glycerol and 0.05% NP-40] for 20 min at room temperature. For the competition reaction, 10-, 20-, 50-, and 100-fold non-labeled probes were mixed with the labeled probes. The reaction mixture was loaded onto a 6% native polyacrylamide gel and run at 4 °C. The DNA shift was detected by developing the biotin signal using the Chemiluminescent Nucleic Acid Detection Module (Thermo Fisher Scientific, USA) according to the manufacturer’s instructions.

### Dual luciferase transcriptional activity assay in rice protoplasts

To test the transcriptional activity of the OsLFL1 protein, the coding sequence of *OsLFL1* was fused in-frame with the GAL4 DNA-binding domain GAL4BD as the effector vector, with CaMV35S-Gal4-LUC as the reporter. To test the transcriptional activation activity of OsLFL1 on *OsMFT1*, the coding sequence of *OsLFL1* was driven by CaMV35S as an effector with the luciferase driven by the promoter of *OsMFT1* as a reporter. The effectors and corresponding reporters were co-transformed into rice protoplasts with the internal control vector CaMV35S-LUC as previously described (Xie and Yang, 2013). The Dual-Luciferase Reporter Assay System (Promega) was used to measure the luciferase activity. Briefly, the rice protoplasts were lysed with Passive Lysis buffer after incubation overnight. The supernatant of the lysate was incubated with luciferase assay substrate and the firefly luciferase (fLUC) activity was measured with the TECAN Infinite M200 System. After the measurement of fLUC, Stop & Glo substrate buffer was added to the reaction and then the *Renilla* luciferase (rLUC) activity was measured. Three independent transformations for each combination were performed, and the relative luciferase activity was calculated by the ratio fLUC/rLUC.

### RNA *in situ* hybridization

The probes for hybridization were amplified from the *OsMFT1* coding sequence using specific primers and inserted into the pGEM-T vector (Promega) for RNA transcription *in vitro*. The respective sense and antisense probes were produced using SP6 and/or T7 transcriptase labelled with the Digoxigenin RNA labeling kit (Roche). Young panicle tissues were collected and fixed in FAA solution (50% ethanol, 5% acetic acid and 3.7% formaldehyde) at 4 °C overnight. RNA *in situ* hybridization and immunological detection were performed as previously described (Zhao *et al.*, 2009).

### Seed germination test

ZH11 and the transgenic homozygous OX-*OsMFT1* lines were grown during normal growing seasons in Wuhan. We marked the panicles when they appeared from the leaf sheath, then harvested the panicles 40 d after their heading. After being dried under sunlight for 3 d, grains were threshed and used for a germination test. Fully filled grains were spread on plates with wet filter paper and immediately moved into an incubator in the dark at 28 °C. Each plate was filled with 50 seeds, and three plates were used for each genotype. Germination was defined as the emergence of the radical, and the number of germinated seeds was counted every half-day after imbibition.

## Results

### *OsMFT1* overexpression and knockout plants showed altered heading date and panicle architecture in rice

**Fig. 1.**
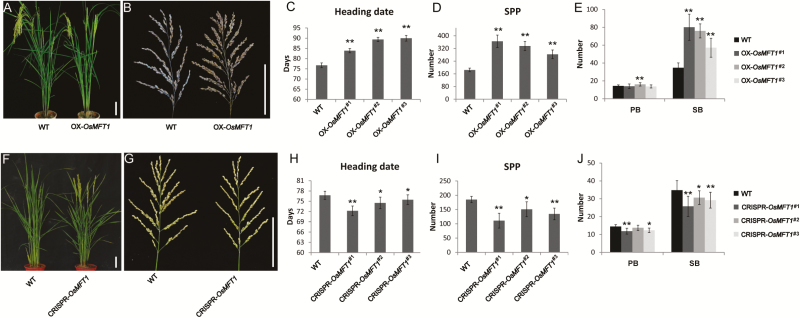
Phenotypes of *OsMFT1* overexpression and knockout transgenic plants grown in the field in summer (long-day condition) in Wuhan. (A, B) Phenotypes of *OsMFT1* overexpression lines (right) and wild type (left) whole plant (A) and panicle (B). Scale bars, 10 cm. (C–E) Comparison of three T_1_ overexpression lines with the wild type for heading date (C), spikelet number per panicle (SPP) (D), primary branches (PB) and secondary branches (SB) (E). (F, G) Phenotypes of *OsMFT1* CRISPR plants (right) and wild type (left) whole plant (F) and panicle (G). Scale bars, 10 cm. (H–J) Comparison of three T_1_ CRISPR lines with the wild type for heading date (H), SPP (I), PB, and SB (E). Error bars indicate the standard deviation (SD), *n*≥15 each. *P<0.05, **P<0.01, *t*-test.

To identify the function of *OsMFT1*, we overexpressed *OsMFT1* using the CaMV35S promoter in a japonica variety Zhonghua 11 (ZH11) and obtained 50 T_0_ transgenic plants, of which 18 were positive (see Supplementary Fig. S1). The positive plants showed delayed heading date and increased spikelet number per panicle compared with wild type (WT) (Fig. 1A, B). Overexpression of *OsMFT1* (OX-*OsMFT1*) in the T_1_ generation significantly delayed heading date by over 7 d and almost doubled spikelet number per panicle (Fig. 1C, D). Overexpression lines had greatly increased number of branches, especially secondary branches (Fig. 1E), resulting in dense panicles. Three lines of *OsMFT1* knockout mutants each having a 1 bp deletion, and 1 bp and 2 bp insertion in the first exon were generated using a CRISPR–Cas9 strategy (Supplementary Fig. S2). A slight but significant promotion in heading date and decrease of spikelets per panicle were observed in all *OsMFT1* knockout mutants compared with WT (Fig. 1F, I). The number of primary branches and secondary branches was significantly reduced (Fig. 1J). Taken together, *OsMFT1* is a suppressor of heading and positive regulator of spikelets per panicle in rice.

### Expression characterization of *OsMFT1* and subcellular localization

**Fig. 2.**
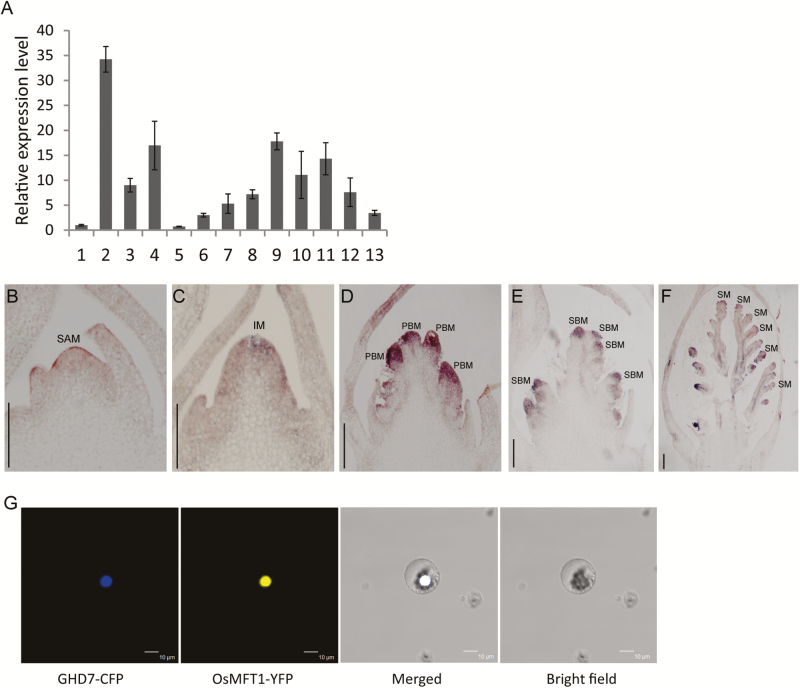
Expression pattern and subcellular localization of *OsMFT1.* (A) RNA expression level of *OsMFT1* in Zhonghua11 in 13 different tissues, including (1) root, (2) stem, (3) sheath, (4) leaf of 30-day plant, (5) 1–2 mm young panicles, (6) 2–5 mm young panicles, (7) 0.5–1 cm young panicles, (8) 1–3 cm young panicles, (9) 3–5 cm young panicles, (10) leaf at 1–2 mm panicle stage, (11) sheath at 1–2 mm panicle stage, (12) leaf at 5 cm panicle stage, and (13) sheath at 5 cm panicle stage. Error bars indicate SD based on three biological replicates. (B–F) RNA *in situ* hybridization analysis of *OsMFT1* expression in the shoot apical meristem (B), inflorescence meristem (C), young panicle at primary branch initiation stage (D), secondary branch initiation stage (E), and spikelet meristem differentiation stage (F). Scale bars, 100 µm. IM, inflorescence meristem; PBM, primary branch meristem; SAM, shoot apex meristem; SBM, secondary branch meristem; SM, spikelet meristem. (G) OsMFT1 colocalized with the transcription factor GHD7 in the nucleus of rice protoplasts.

To characterize the spatial–temporal expression pattern of *OsMFT1*, the RNA transcript level of *OsMFT1* was examined in roots, stems, leaves, sheaths, and developing young panicles using quantitative real-time PCR. *OsMFT1* was preferably expressed in stem, leaves, sheath, and developing young panicles (Fig. 2A). RNA *in situ* hybridization revealed that *OsMFT1* was slightly expressed in the shoot apical meristem and inflorescence meristem (Fig. 2B, C), and strongly expressed in the primary branch meristem (Fig. 2D), secondary branch meristem (Fig. 2E) and spikelet meristem (Fig. 2F). To determine the subcellular localization of OsMFT1, the full length coding sequence (CDS) of *OsMFT1* was fused to the yellow fluorescent protein (YFP) reporter gene driven by the CaMV 35S promoter. Then, the OsMFT1-YFP and GHD7-CFP plasmids were co-transformed into protoplasts. The OsMFT1-YFP fusion protein was luminescent in the nucleus and the YFP fluorescence overlapped with cyan fluorescent protein (CFP) fluorescence, which indicated that OsMFT1 co-localized with GHD7, a nuclear protein (Fig. 2B). Thus, OsMFT1 is a nuclear protein.

### The role of *OsMFT1* in regulating heading date

**Fig. 3.**
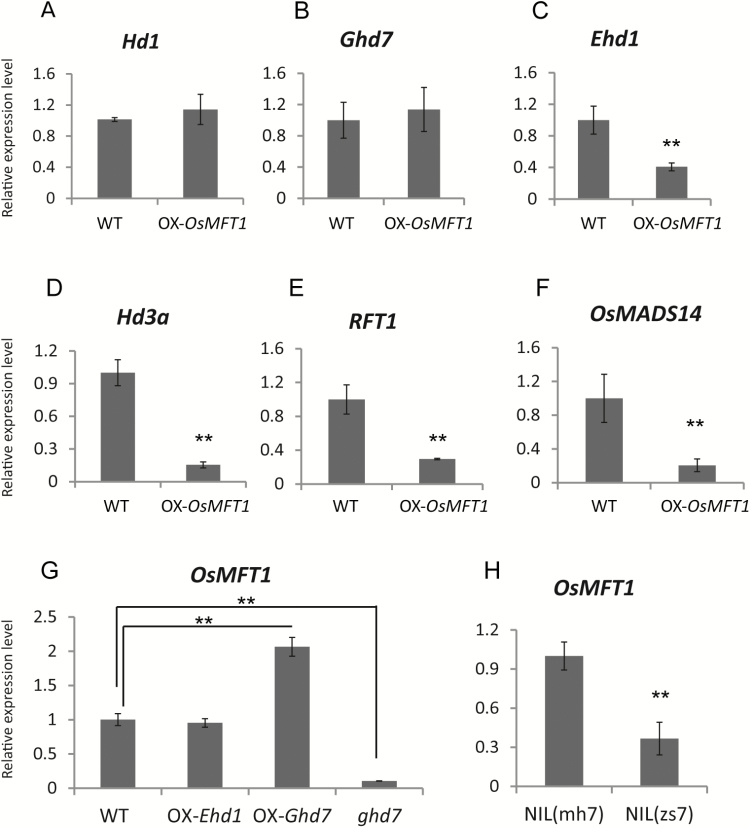
Transcriptional regulation between *OsMFT1* and the key flowering genes. (A–F) mRNA expression comparison of *OsMFT1*, *Ghd7*, *Ehd1*, *Hd3a*, *RFT1*, and *MADS14* between wild type and *OsMFT1* overexpression lines. (G) Expression of *OsMFT1* in Zhonghua 11 (ZH11), *Ehd1* overexpression lines (OX-*Ehd1*), *Ghd7* overexpression lines (OX-*Ghd7*) and *Ghd7* knockout mutant (ghd7). (H) Expression of *OsMFT1* in *Ghd7* near isogenic lines (NILs). NIL(mh7) had a strongly functional allele of *Ghd7* while NIL(zs7) had a non-functional *Ghd7*. Plants were grown in a growth chamber (14 h light/10 h dark cycle for LD conditions) and the top–second leaves of 45-day plants were sampled for RNA extraction at 2 h after lights on when most flowering genes reached their peaks of RNA expression level. Error bars indicate SD based on three biological replicates. **P<0.01, *t*-test.

*OsMFT1* overexpression lines and wild type were used to examine the transcriptional level of key genes involved in the photoperiodic flowering pathway. There were no significant differences in the expression of *Ghd7* and *Hd1* between *OsMFT1* overexpression lines and wild type, while the expression levels of *Ehd1*, *Hd3a*, *RFT1*, and *MADS14* were greatly suppressed in overexpression lines (Fig. 3A–G), indicating that *OsMFT1* acted upstream of *Ehd1*. To further elucidate the regulatory relationship between *OsMFT1* and these genes, the RNA expression level of *OsMFT1* in an *Ehd1* overexpression line (OX-*Ehd1*), a *Ghd7* overexpression line (OX-*Ghd7*), a *Ghd7* mutant line (*ghd7*), and a pair of *Ghd7* near isogenic lines was examined. Compared with the wild type ZH11, the expression level of *OsMFT1* did not vary in the OX-*Ehd1* line, indicating that *Ehd1* did not regulate *OsMFT1* in turn (Fig. 3G). The expression level of *OsMFT1* was approximately 4-fold more than that in the OX-*Ghd7* line and was reduced by 3-fold in the *Ghd7* mutant compared with wild type (Fig. 3G). Similarly, the expression level of *OsMFT1* in NIL(mh7) was 3-fold of that in NIL(zs7), which has completely lost *Ghd7* (Fig. 3H). In general, *OsMFT1* expression is regulated by *Ghd7* but not by *Ehd1* and, in contrast, *OsMFT1* suppressed *Ehd1* expression. The expression of other flowering genes was also examined in *OsMFT1* overexpression lines and wild type, but no significant differences were detected (see Supplementary Fig. S3). Thus, it is suggested that *OsMFT1* acts downstream of *Ghd7* and upstream of *Ehd1* in the photoperiod flowering pathway.

### *OsMFT1* overexpression rescued the phenotype of Ami-*Ghd7*

**Fig. 4.**
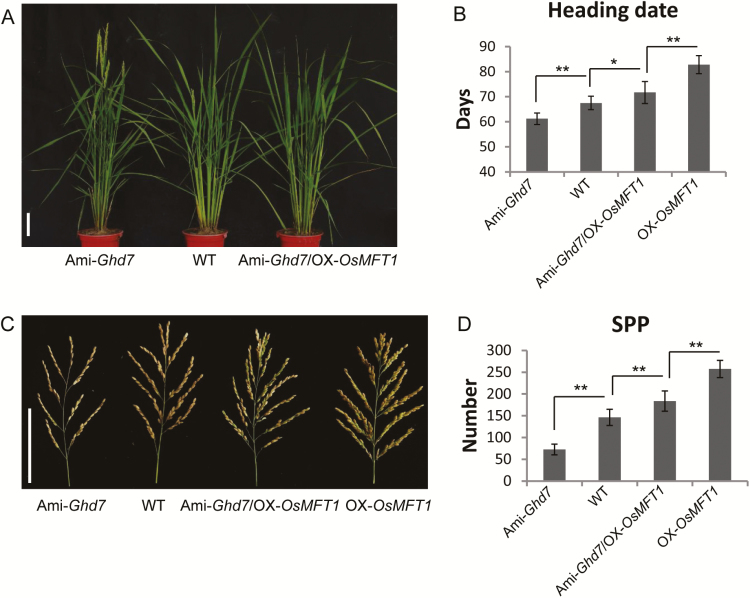
Comparison of heading date and panicle architecture among wild type, single mutants, and double mutants in LD condition. (A, B) Heading date of Ami-*Ghd7* (artificial microRNA-mediated *Ghd7* silencing in ZH11), OX-*OsMFT1* (*OsMFT1* overexpressing lines), their hybrid Ami-*Ghd7*/OX-*OsMFT1* and WT. (C, D) Panicle architecture of Ami-*Ghd7*, OX-*OsMFT1*, Ami-*Ghd7*/OX-*OsMFT1*, and WT. SPP, spikelets per panicle. Error bars indicate SD; *n*≥10 for each.

To understand the effect of *OsMFT1* on *Ghd7*-mediated flowering and panicle architecture, a hybrid F_1_ was generated by crossing OX-*OsMFT1* with Ami-*Ghd7* (*Ghd7* artificial microRNA), in which *Ghd7* expression was largely suppressed. Four genotypes showing higher *OsMFT1* and lower *Ghd7* (Ami-*Ghd7*/OX-Os*MFT1*), higher *OsMFT1* but normal *Ghd7* (OX-*OsMFT1*), lower *Ghd7* but normal *OsMFT1* (Ami-*Ghd7*) and wild type were identified from an F_2_ population (see Supplementary Fig. S4). The heading date and spikelets per panicle of four genotypes in long-day conditions are displayed in Fig. 4. Compared with the wild type plants, Ami-*Ghd7* showed significantly advanced heading date and smaller panicle size while the *OsMFT1* overexpression line (OX-*OsMFT1*) showed significantly delayed heading date and denser panicle architecture. The double mutant, Ami-*Ghd7*/OX-Os*MFT1*, simultaneously overexpressing *OsMFT1* and suppressing *Ghd7*, exhibited a 4-day delay in heading date and an approximately 25% increase in spikelets per panicle than the wild type. Similar differences in phenotypes among four genotypes were observed in SD conditions, but the genotypes had smaller phenotype values than their corresponding phenotype values in the LD conditions (Supplementary Fig. S5). These results indicated that *OsMFT1* overexpression rescued the phenotype of Ami-*Ghd7* in heading date and spikelets per panicle, and *OsMFT1* was indeed acting downstream of *Ghd7*.

### OsLFL1 activated *OsMFT1* expression by directly binding to its promoter

**Fig. 5.**
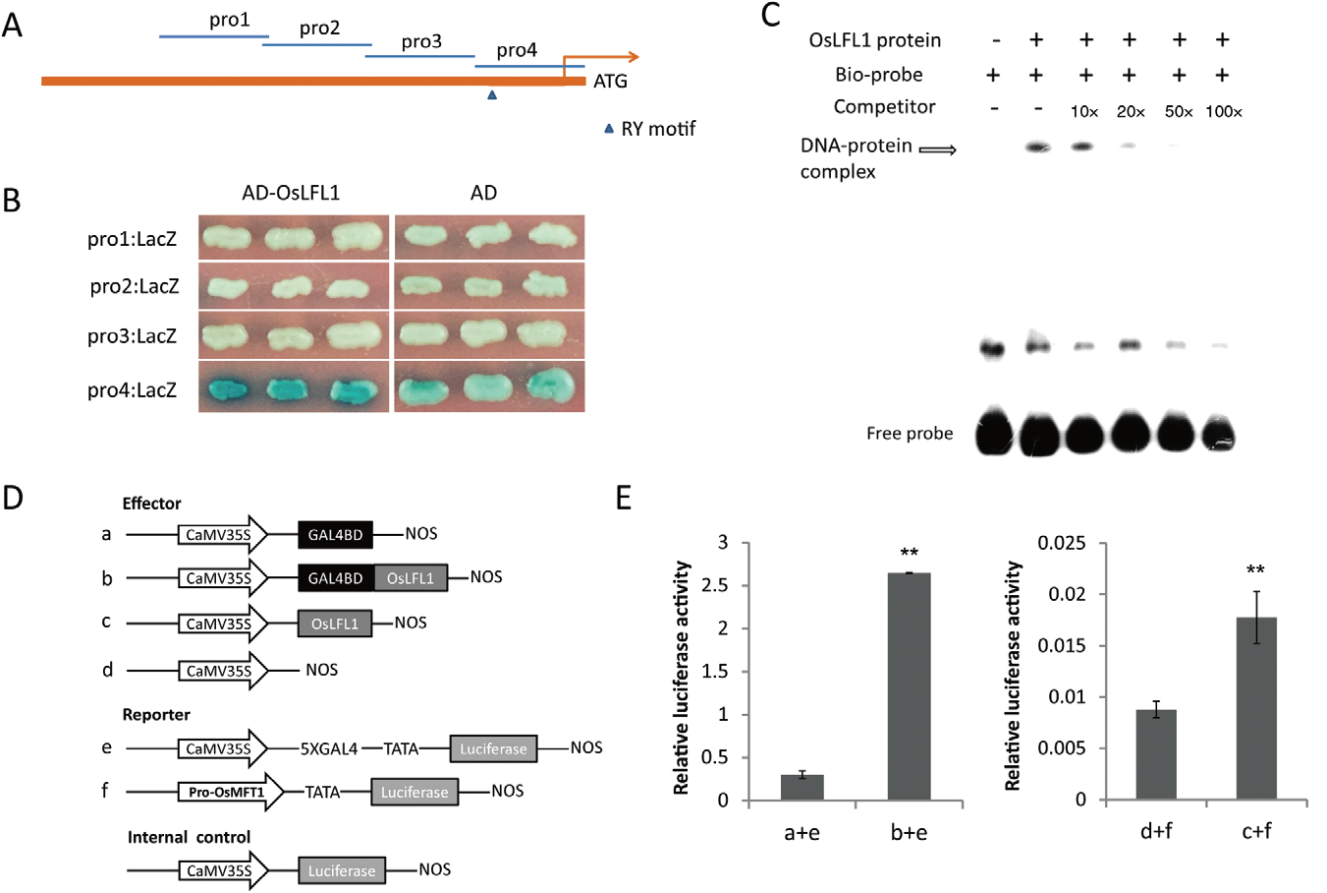
*In vivo* and *in vitro assay* of OsLFL1 binding to the promoter of *OsMFT1.* (A) The promoter of *OsMFT1* was divided into four fragments (pro1–4), and the RY motif was contained in pro4. (B) OsLFL1 bound to pro4 in yeast cells through a yeast one-hybrid assay on selective medium (SD/−Trp−Ura) containing X-gal for developing the blue color. (C) EMSA assay using the OsLFL1 protein and 50-bp *OsMFT1* promoter containing RY motif as a probe labeled with 5′-biotin. The 10-, 20-, 50- and 100-fold non-labeled probes were used for competition. (D, E) OsLFL1 activates the expression of *OsMFT1* by dual luciferase transient assay in rice protoplasts. Error bars indicate SD based on three biological replicates, **P<0.01, *t*-test.

In Arabidopsis, ABI3 directly binds to the RY motif in the promoter of *AtMFT* (Park *et al.*, 2011; Mao and Sun, 2015). Among the ABI3 homologs reported in rice, *OsLFL1* is a flowering repressor (Peng *et al.*, 2008; Romanel *et al.*, 2009). Thus, OsLFL1 is proposed to probably bind to *OsMFT1* promoter directly. Then, the 1.6 kb sequence of the *OsMFT1* promoter and 5′-UTR region was divided into four fragments and used to perform a yeast one-hybrid assay with OsLFL1 protein. A binding activity of OsLFL1 protein to the fourth fragment (pro4) closest to ATG was identified (Fig. 5A, B). *cis*-Element analysis identified an RY motif (CATGCATG) 221 bp upstream of the translation start site ATG in the *OsMFT1* promoter (Fig. 5A). An electrophoretic mobility shift assay (EMSA) showed that OsLFL1 protein directly bound to the 50-bp fragment containing the RY motif *in vitro* (Fig. 5C). To verify how OsLFL1 regulates *OsMFT1*, a dual-luciferase transient assay was performed in rice protoplasts to examine the transcriptional activity of *OsLFL1*. As shown in Fig. 5D, E, with the firefly luciferase driven by five copies of the yeast GAL4 binding domain (GAL4BD) as a reporter, relative luciferase activity of OsLFL1 fused with GAL4BD as an effector was much higher than GAL4BD itself as an effector, indicating that OsLFL1 had significant transcriptional activation activity; with the firefly luciferase driven by the *OsMFT1* promoter as a reporter, relative luciferase activity of OsLFL1 as an effector was 2-fold of empty ‘none’ as an effector indicating that OsLFL1 had activation activity on the *OsMFT1* promoter. Taken together, OsLFL1 activates *OsMFT1* expression by directly binding to its RY motif in the promoter.

### Overexpression of *OsMFT1* suppressed the expression of spikelet meristem identity genes

**Fig. 6.**
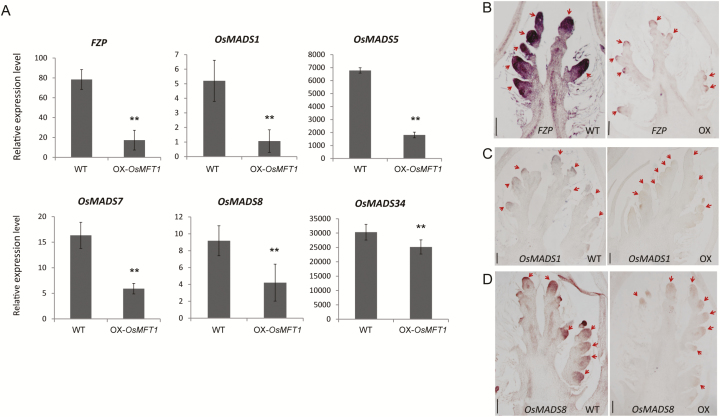
Differential expression of rice *SEP*-like genes and *FZP* between *OsMFT1* overexpression lines and wild type. (A) RNA expression comparison of *FZP*, *OsMADS1*, *OsMADS5*, *OsMADS7*, *OsMADS8*, and *OsMADS34* between OX-*OsMFT1* and wild type using quantitative real time PCR. Error bars indicate SD based on three biological replicates. (B–D) RNA *in situ* hybridization of *FZP* (B), *OsMADS1* (C), and *OsMADS8* (D) in young panicles of wild type (left) and *OsMFT1* overexpression lines (right). Scale bars, 100 µm. Red arrowheads show secondary branch meristems.

To identify downstream genes of *OsMFT1*, the 0.5–1 mm young panicles from OX-*OsMFT1* and wild type plants were used for transcriptome sequencing to find differentially expressed genes. Compared with wild type, 84 genes were up-regulated and 86 genes were down-regulated in the OX-*OsMFT1* young panicles (see Supplemenatry Table S2). Three genes regulating spikelet meristem differentiation were down-regulated, including *MADS1*, *MADS5*, and *FZP*. *FZP* is a spikelet meristem identity gene that determines the transition from panicle branching to spikelet formation. *OsMADS1* and *OsMADS5*, together with *OsMADS7*, *OsMADS8*, and *OsMADS34* are five *SEPALLATA*-like genes that are classified as class E genes for floral determinacy (Cui *et al.*, 2010). A qRT-PCR assay showed that these genes were indeed down-regulated in OX-*OsMFT1* (Fig. 6A). Further RNA *in situ* hybridization showed that the significantly weaker expression of *FZP*, *OsMADS1*, and *OsMADS8* was detected in the secondary branch meristem of OX-*OsMFT1* compared with wild type (Fig. 6B–D).

## Discussion

### Conserved functions in regulating flowering and panicle architecture between *MFT*-like and *TFL1*-like families in rice

Most of the PEBP family genes are functionally conserved among higher plants. Previous studies indicated that *FT*-like genes generally induce flowering both in monocots and dicots, while *TFL1*-like genes delay flowering and regulate the inflorescence architecture, including *TFL1* in Arabidopsis, *RCNs* in rice, and *ZCNs* in maize (Alvarez *et al.*, 1992; Nakagawa *et al.*, 2002; Zhang *et al.*, 2005; Danilevskaya *et al.*, 2010; Wickland and Hanzawa, 2015). As the evolutionary ancestor of *FT* and *TFL1*, *MFT*-like genes are generally related to seed germination (Xi *et al.*, 2010; Nakamura *et al.*, 2011; Li *et al.*, 2014; Hu *et al.*, 2016). As expected, *OsMFT1* also regulates seed germination, which will be further studied in future (see Supplementary Fig. S6). *OsMFT1* overexpression significantly delays flowering and largely increases branching, and the *OsMFT1* knockout mutant promotes flowering and reduces branching. The performance of *OsMFT1* overexpression plants is very similar to rice *TFL1*-like gene (*RCN1*, *RCN2*, and *RCN3*) overexpression plants (Nakagawa *et al.*, 2002; Zhang *et al.*, 2005). In addition, knocking down four *TFL1*-like genes in rice resulted in small panicles with reduced branches, similar to the *OsMFT1* knockout plants (Liu *et al.*, 2013). We examined the expression of rice *TFL1*-like genes (*RCN1*, *RCN2*, and *RCN3*) in the leaves and panicles of *OsMFT1* overexpression lines and wild type, and no big differences were detected (Supplementary Fig. S7). Since *OsMFT1* and rice *TFL1*-like genes have similar functions in regulating flowering and inflorescence architecture, functional redundancy may exist between them.

FT and TFL1 have only 39 non-conservative amino acid substitutions but have distinct functions due to a potential ligand binding residue and a divergent external loop (Hanzawa *et al.*, 2005; Ahn *et al.*, 2006). Lateral studies revealed that the mutation of at least four residues converts FT into a complete TFL1 mimic by affecting the protein surface charge through testing the effects of numerous mutations of FT *in vivo* (Ho and Weigel, 2014). We compared the crucial amino acid residues of FT-like and TFL1-like protein in both Arabidopsis and rice (see Supplementary Fig. S8); furthermore, we analyzed the potential function of OsMFT1 according to the reported mutations in FT and their corresponding phenotypes (Ho and Weigel, 2014). The results indicate that the key amino acid residues of FT-like and TFL1-like proteins are conserved between Arabidopsis and rice, and the methionine (M) at position 109, lysine (K) at 128 and alanine (A) at 138 most likely contribute to the conferred TFL1 activity of OsMFT1 (Supplementary Table S3), which could explain the functional similarity of *OsMFT1* and *TFL1*-like genes in rice.

In Arabidopsis, *MFT* mainly functions in regulating seed germination instead of flowering (Yoo *et al.*, 2004; Xi *et al.*, 2010). In this study, *OsMFT1* had effects on both seed germination and flowering (Fig. 1; Supplementary Fig. S6). It is noted that the upstream regulator *Ghd7* and downstream gene *Ehd1* of *OsMFT1* are specific genes in rice that have no homologs identified in Arabidopsis (Doi *et al.*, 2004; Xue *et al.*, 2008). This is probably why *MFT1* has no regulation in flowering in Arabidopsis.

### Activation of *OsMFT1* by both OsLFL1 and *Ghd7*

A previous study has proposed that *Ghd7* positively regulates *OsMFT1* in flowering through an eQTL-guided function-related co-expression analysis (Wang *et al.*, 2014). Here, we have further demonstrated that *Ghd7* regulates *OsMFT1* expression transcriptionally and *OsMFT1* plays a role in regulating flowering time and spikelets per panicle downstream of *Ghd7* using double mutants. Ghd7 is a central regulator that has many downstream targets to regulate multiple traits (Weng *et al.*, 2014), and *OsMFT1* is only one of the downstream targets of *Ghd7*. That is probably why Ami-*Ghd7*/OX-*MFT1* did not fully show the performance of OX-*OsMFT1* and exhibited an intermediate phenotype. Both OsLFL1 and GHD7 are transcriptional factors activating *OsMFT1* expression at transcriptional level. OsLFL1 directly binds to the promoter of *OsMFT1* (Fig. 5), but yeast one-hybrid assay demonstrated that GHD7 didn’t bind to the *OsMFT1* promoter (see Supplementary Fig. S9), indicating that GHD7 probably regulates *OsMFT1* expression indirectly. Recent studies have revealed that an RY motif in *FLC* and *EUI1* could recruit a complex for gene repression (Yuan *et al.*, 2016; Xie *et al.*, 2018). It is possible that an RY motif in OsMFT1 recruits a protein complex consisting of OsLFL1 and GHD7 for gene activation, or one of the *Ghd7* downstream targets also directly binds to the promoter of *OsMFT1*.

### Prolonged branch meristem differentiation caused dense panicles in *OsMFT1* overexpression plants

Rice panicle is derived from the inflorescence meristem, and the inflorescence meristem develops branch meristems. The differentiation of spikelet meristems indicates that the branch meristem stops producing branch primordia and terminates to be a spikelet. Here, we found that overexpressing *OsMFT1* significantly represses *FZP* and *SEPALLATA*-like genes expression. Repression or absence of *FZP* causes the branch meristem to produce more lateral branch primordia instead of spikelet primordia and overexpression of *FZP* accelerates the spikelet formation, which results in fewer branches (Komatsu *et al.*, 2003; Bai *et al.*, 2016). The latest reports on *FZP* have revealed that transcriptional silencer-mediated repression of *FZP* expression increases spikelet number per panicle (Bai *et al.*, 2017). Class E genes are required for floral determinacy, including the five *SEPALLATA*-like genes *OsMADS1*, *OsMADS5*, *OsMADS7*, *OsMADS8*, and *OsMADS34*. Ectopic expression of *OsMADS1* was reported to cause reduced branches and small panicles (Wang *et al.*, 2017). Thus the delay of accumulation of RNA expression of *FZP* and *SEPALLATA*-like genes indicates the delay of acquiring spikelet meristem identity. Hence, we proposed OX-*OsMFT1* has prolonged branch meristem differentiation, which leads to increased branches and spikelets. Nevertheless, the mechanism of *OsMFT1* regulation of *FZP* and *SEPALLATA*-like genes is still unknown.

**Fig. 7.**
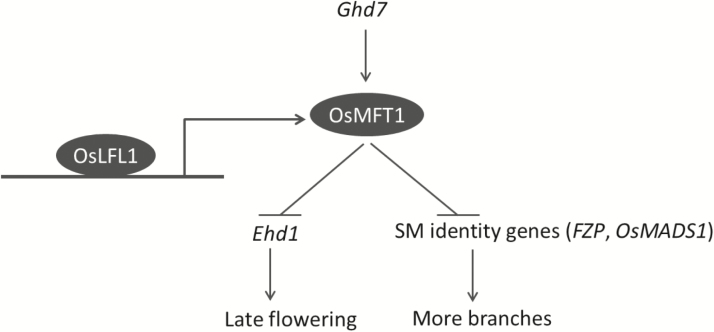
A suggested working model of *OsMFT1.*

Based on these results, we propose a working model of *OsMFT1* (Fig. 7). OsLFL1 protein directly binds to the *OsMFT1* promoter and activates *OsMFT1* expression. *OsMFT1* negatively regulates flowering downstream of *Ghd7* and upstream of *Ehd1*. Meanwhile, *OsMFT1* represses the expression of genes for spikelet meristem identity and prolongs branch differentiation, which results in more panicle branches. Hence, *OsMFT1* is a suppressor of heading and a positive regulator of panicle architecture in rice.

## Supplementary data

Supplementary data are available at JXB online

Fig. S1. RNA expression level of partial T_0_ individuals of OX-*OsMFT1*.

Fig. S2. The mutation positions and mutation types of three *OsMFT1* CRISPR lines.

Fig. S3. RNA expression level comparison of some rice flowering genes.

Fig. S4. Expression of *Ghd7* and *OsMFT1* in four genotypes from an F2 population.

Fig. S5. Comparison of heading date and panicle architecture among four genotypes.

Fig. S6. Comparison of germination speed between ZH11 and OX-*OsMFT1* lines.

Fig. S7. RNA expression level comparison of rice *TFL1*-like genes between ZH11 and OX-*OsMFT1* lines.

Fig. S8. Amino acid alignment of FT-like and TFL1-like protein segments in Arabidopsis and rice.

Fig. S9. Yeast one-hybrid assay of GHD7 and *OsMFT1* promoter.

Table S1. Primers used in this study.

Table S2. Differentially expressed genes between ZH11 andOX-*OsMFT1* young panicles.

Table S3. Comparison of six important amino acid residuesbetween AtFT, AtTFL1, and OsMFT1.

Supplementary Material

Supplementary Table S2
