# Supplementary material for: OsMFT1 increases spikelets per panicle and delays heading date in rice by suppressing Ehd1, FZP and SEPALLATA-like genes
Source: J Exp Bot. 2018 Jun 21;69(18):4283–93. doi: 10.1093/jxb/ery232 (PMC6093437; doi:10.1093/jxb/ery232)
Supplement: Supplementary Material [file ery232_suppl_supplementary_material.pdf]

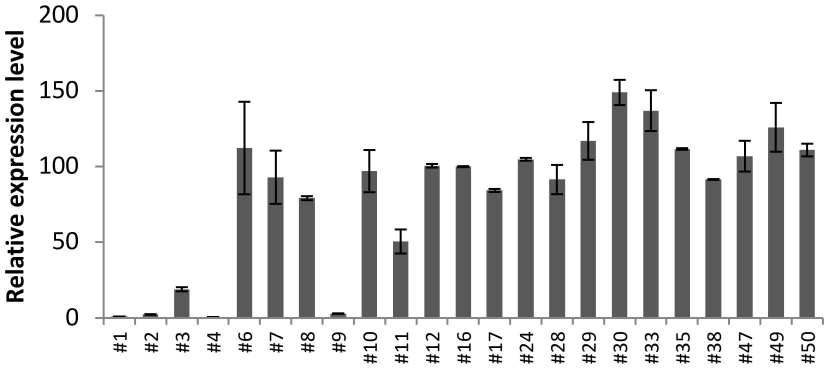

Fig. S1. RNA expression level of partial T_0 individuals of OX-*OsMFT1*. Among these individuals, #1, #2, #3, #4 and #9 displayed wild type phenotypes while the else displayed late flowering and dense panicle. Error bars indicate SD based on three technical replicates.

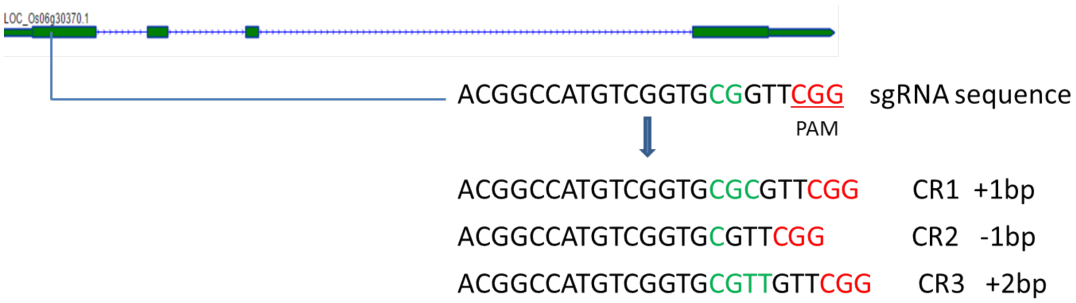

Fig. S2. The mutation positions and mutation types of three *OsMFT1* CRISPR lines

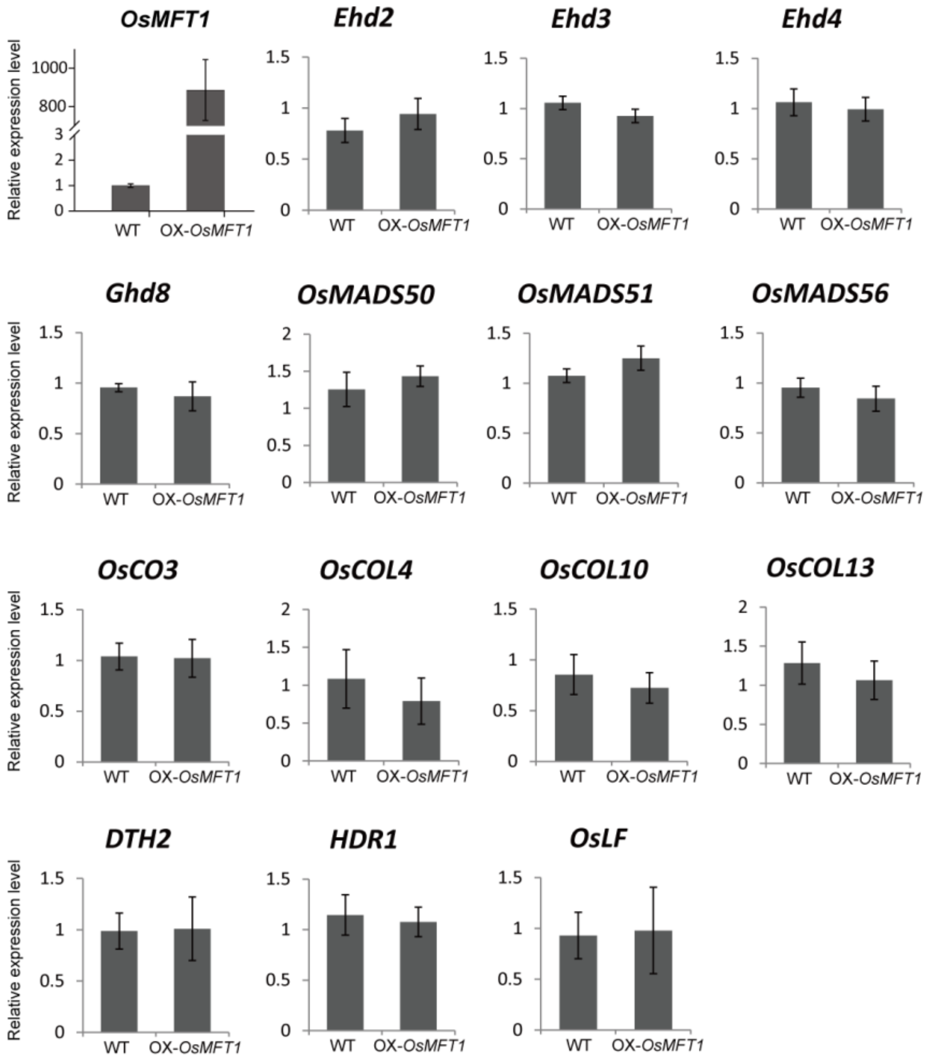

Fig. S3. RNA expression level comparison of some rice flowering genes between wild type and *OsMFT1* overexpression lines.

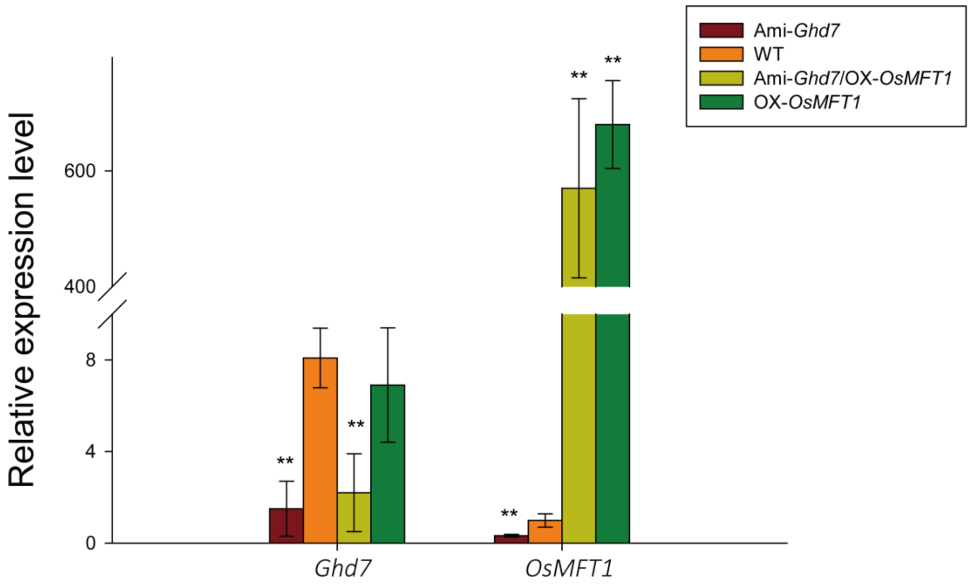

Fig. S4. Expression of *Ghd7* and *OsMFT1* in four genotypes from an F2 population. Error bars indicate SD, n≥10 each. $^{**}P<0.01$ compared with WT, *t*-test.

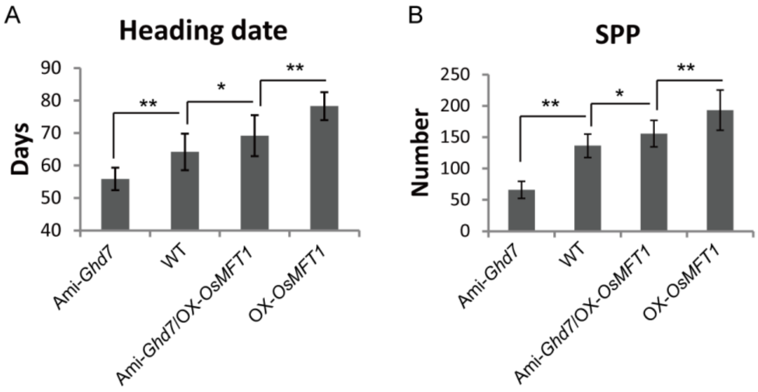

Fig. S5. Comparison of heading date and panicle architecture among wild type, single mutants and double mutants in short-day conditions. Heading date (A) and spikelet number per panicle (B) of Ami-*Ghd7* (artificial microRNA mediated *Ghd7* silencing in ZH11), OX-*OsMFT1* (*OsMFT1* overexpressing lines), their hybrid Ami-*Ghd7*/OX-*OsMFT1* and WT. SPP, spikelet per panicle. Error bars indicate SD, n⩾8 each. $^{*}P < 0.05$, $^{**}P < 0.01$, *t*-test.

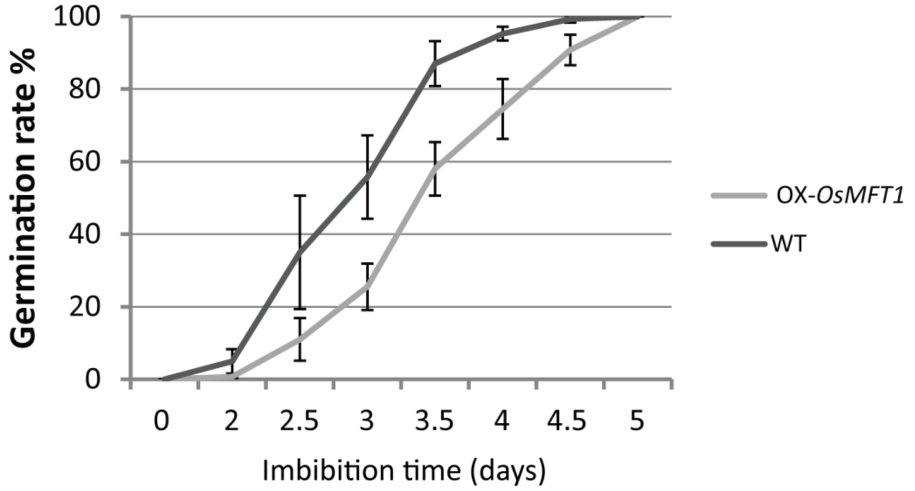

Fig. S6. Comparison of germination speed of the wild-type ZH11 and the *OsMFT1* overexpression lines. Error bars indicate SD based on three biological replicates.

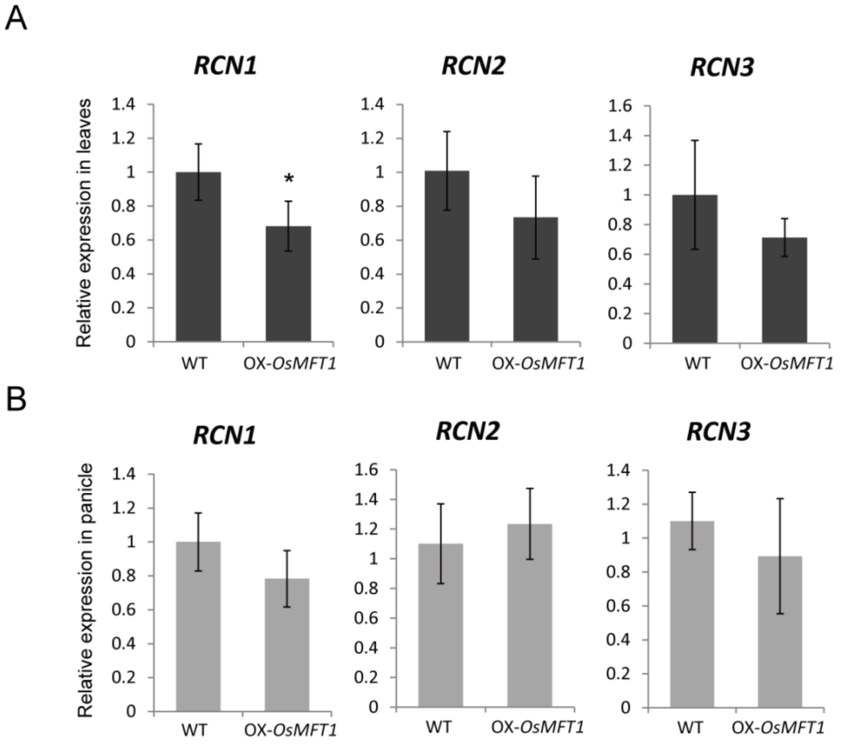

Fig. S7. RNA expression level comparison of rice *TFL1*-like genes between wild-type ZH11 and *OsMFT1* overexpression lines in leaves (A) and 0.5-1 mm young panicles (B). *$P < 0.05$, *t*-test.

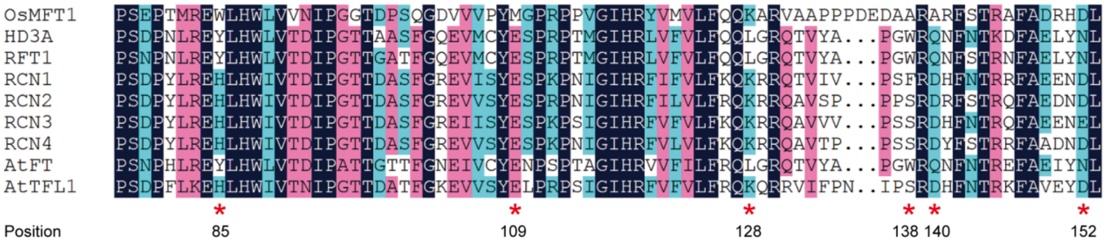

Fig. S8 Amino acid alignment of FT-like and TFL1-like protein segments in both Arabidopsis and rice. The red asterisk indicated the key residues as reported previously. Amino acid positions indicated are based on the Arabidopsis FT protein sequence.

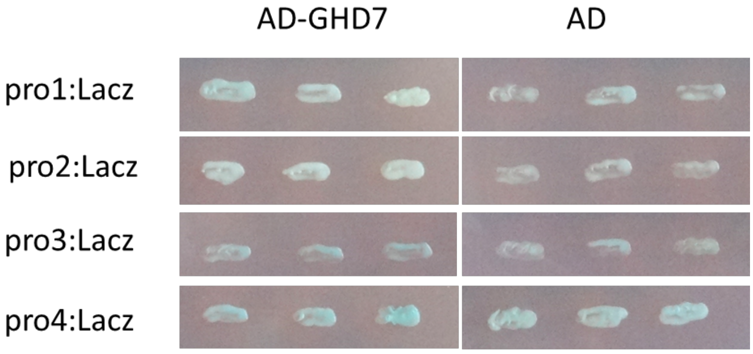

Fig.S9. Yeast one-hybrid assay of GHD7 and *OsMFT1* promoter. The promoter of *OsMFT1* was divided into four fragments (pro1-4) and no binding was detected of GHD7 to the four fragments through a yeast one-hybrid assay on selective medium (SD/-Trp-Ura) containing X-gal for developing the blue color.

Table S1 Primers used in this study

For constructs generation	
OX-OsMFT1-F	GGTACCGCCATGGCATCGCATGTGGA
OX-OsMFT1-R	TCTAGAGACCGATGGCTTATTAGCTAG
CRISPR-OsMFT1-F	ACGGCCATGTCGGTGCGGTTGTTTTAGAGCTAGAAATAGCAAGTTA
CRISPR-OsMFT1-R	AACCGCACCGACATGGCCGTGCCACGGATCATCTGCACAAC
EYFP-MFT1-XbaI-F	CTCTAGCAGATCTATCGATTCTAGAATGGCATCGCATGTGGACCC
EYFP-MFT1-XbaI-R	CTTGCTCACCATGGCTCTAGAGTAGCGGCGGCGGCGGTTGG
190LUC-proOsPRO-MFT1-F	CATTTGGAGAGGACACGCTGGATCCGCCATTCATCACCCGATACCA
190LUC-proOsPRO-MFT1-R	TTGTTGTTGTTGGTAATTGTGGATCCTAAGCTCTCGCGGTGACTCT
35SNONE-OsLFL1-F	CCCCCGGGCTGCAGGAATTCATGCGGGGGGAGGAGAGATG
35SNONE-OsLFL1-R	ATAAGCTTGATATCGAATTCTCACATGTGAGGCCCAGACT
Gal4DBD-OsLFL1-F	CCCCCGGGCTGCAGGAATTCATGCGGGGGGAGGAGAGATG
Gal4DBD-OsLFL1-R	ATAAGCTTGATATCGAATTCTCACATGTGAGGCCCAGACT
Y1H-AD-OsLFL1-F	TGCCAGATTATGCCTCTCCCGAATTCATGCGGGGGGAGGAGAGATG
Y1H-AD-OsLFL1-R	CAAAGCTTCTCGAGTCGGCCGAATTCTCACATGTGAGGCCCAGACT
Y1H-lacz-PRO-MFT1-1F	GTACCCGGGGATCTGTCGACCTCGAGCATCTTCTCTGCTGGCCTCC
Y1H-lacz-PRO-MFT1-1R	TATACATACAGAGCACATGCCTCGAGTTGCTTGACATTTGTGAGGG
Y1H-lacz-PRO-MFT1-2F	GTACCCGGGGATCTGTCGACCTCGAGCCCTCACAAATGTCAAGCAA
Y1H-lacz-PRO-MFT1-2R	TATACATACAGAGCACATGCCTCGAGAAAAGATCCATCAAGATAAC
Y1H-lacz-PRO-MFT1-3F	GTACCCGGGGATCTGTCGACCTCGAGGCAGAGGTTCTATTCTTGTA
Y1H-lacz-PRO-MFT1-3R	TATACATACAGAGCACATGCCTCGAGTTTACATGCTAAAACTGCCT
Y1H-lacz-PRO-MFT1-4F	GTACCCGGGGATCTGTCGACCTCGAGCAGTTCACAAATTAAGAGAT
Y1H-lacz-PRO-MFT1-4R	TATACATACAGAGCACATGCCTCGAGCGTTGGTGAGGTCCTTGGT
EMSA-probe-F	ACACAATCTCCAAATTAAAACCATGCATGCTTATCATCATCACCATCACC
EMSA-probe-R	GGTGATGGTGATGATGATAAGCATGCATGGTTTTAATTTGGAGATTGTGT
LFL1-protein-F	AATAAACGCTCAACTTTGGCAGATCTATGCGGGGGGAGGAGAGATG
LFL1-protein-R	CTCACTATAGGGAGACCGGCAGATCTTCACATGTGAGGCCCAGACT
In situ probe_MFT1_F	GACGACGGCCATGTCGGTGC
In situ probe_MFT1_R	AAGCCACTCTCTCATAGTCG
In situ probe_OsMADS1_F	GAAGAGCAAGGAGCAACAGC
In situ probe_OsMADS1_R	GCCTGAAGCCTGAACTGAAC
In situ probe_OsMADS8_F	GATCAATGCCCAGCACTACC
In situ probe_OsMADS8_R	AGGCTGATGCATGATGTTGA
In situ probe_FZP_F	ATGAACACTCGAGGCAGC
In situ probe_FZP_R	CTCGCTCATCGGCGACGA
For real time PCR	
qrt-Ubq-F	AACCAGCTGAGGCCCAAGA
qrt-Ubq-R	ACGATTGATTTAACCAGTCCATGA
qrt-Ehd1-F	TGGAAATCTCGAAAAACCCG
qrt-Ehd1R	GCGCTAGCAAAGCTTCGGT
qrt-RFT1-F	TGACCTAGATTCAAAGTCTAATCCTT

qrt-RFT1-R	TGCCGGCCATGTCAAATTAATAAC
qrt-Hd3a-F	GCTCACTATCATCATCCAGCATG
qrt-Hd3a-R	CCTTGCTCAGCTATTTAATTGCATAA
qrt-Ghd7-F	AGGTGCTACGAGAAGCAAATCC
qrt-Ghd7-R	GGGCCTCATCTCGGCATAG
qrt-OsMADS14-F	TGGGACCAGACACAACCTCA
qrt-OsMADS14-R	CCTGCTGCTACATCCTCTAT
qrt-OsMADS1-F	ATCACCATCAGGGTCTTCTC
qrt-OsMADS1-R	CAACCATGTCTGCTGCTTCA
qrt-OsMADS5-F	GCTTCATATATCTTGCCAAG
qrt-OsMADS5-R	TTGGTTGAGGTGATCCATGT
qrt-OsMADS7-F	TGGGTTCTTCCATCCACTTG
qrt-OsMADS7-R	CGTCATCATCATGGTAGCCA
qrt-OsMADS8-F	CACCTTGCAGATCGGGTTTA
qrt-OsMADS8-R	ATCTGTGTCGTCACATCCGT
qrt-OsMADS34-F	CAACCAGAGCACTTCTTCCA
qrt-OsMADS34-R	CTGAAGCTGAAACGGTAGCT
qrt-FZP-F	GCCACGACTTCCTCTTCTC
qrt-FZP-R	GCCGCATGTCCTGATGAT
qrt-RCN1-F	GGTGACCTGCGATCTTTCTT
qrt-RCN1-R	CAATGAAGGTGCTCCCTTAGAT
qrt-RCN2-F	GGAGCATCTTCACTGGATTGT
qrt-RCN2-R	CCTGTGGATGCCGATGTT
qrt-RCN3-F	CCAGGACCAAGTGATCCATATC
qrt-RCN3-R	AGCTTATGATTTCCCGTCCAA
qrt-Hd1-F	TCAGCAACAGCATATCTTTCTCATCA
qrt-Hd1-R	TCTGGAATTTGGCATATCTATCACC
qrt-OsMFT1-F	TCAACGAGCTCTTCGCTCTG
qrt-OsMFT1-R	ATGTACGGCACCACCACATC
qrt-Ghd8-F	CGTGCAATGGTTTAGACTAAAG
qrt-Ghd8-R	AACAGCATCAGCATCAACAA
qrt-Ehd2-F	AGCTGAAGAGAGTGCAAGGCTTCT
qrt -Ehd2-R	TGATGTTGCTGTTGTTGCAGGTGG
qrt-Ehd3-F	CAAACACTGTGGCACATGCGAAGA
qrt -Ehd3-R	TCATCCTTGTCTTGGAAGCACCCT
qrt-Ehd4-F	CAGCCAGCGGAATCATCAC
qrt –Ehd4-R	CCAAATCCATCAGACCTACTCCT
qrt-OsMADS50-F	GACCGTAACATCAACACCAC
qrt - OsMADS50-R	GAGATCCAGCTTATTCCTGG
qrt- OsMADS51-F	GAAATCAAAGAAGATGTTGGCAAA
qrt - OsMADS51-R	CTTCCTCCTGCCCCCTAGAG
qrt- OsMADS56-F	AGCTCTTGATGAGTCCAGACGGAA
qrt - OsMADS56-R	AGTCCGCTCCTTCTCTTTCAGCTT

qrt-CO3-F	GGAGAAGAGGAAGACGAGGC
qrt -CO3-R	TAGCTAAGCAACCAAGATGTA
qrt-COL4-F	ATGCGGATGATGACCGGAGCAA
qrt –COL4-R	TATCCTCGTGGCTATTGTCGTCGG
qrt-COL13-F	GAGTTCACTTCTGGGCATGGTG
qrt –COL13-R	TCAGTGCAATCGGTTGACATGA
qrt-COL10-F	CGCCCTCGCTTCGATCCCA
qrt –COL10-R	TCCCTCTCGCCGCCGGTCA
qrt-DTH2-F	CCAGTTTCAACGACGCCTAA
qrt-DTH2-R	GTCTCCATATACGCTCCCATCA
qrt-OsLF-F	AACCCTAGGGAATGGCAATG
qrt-OsLF-R	CGCCCAAATGCAAGTACAGT
qrt-HDR1-F	TGGTTGGCAAGAAACGCAAG
qrt-HDR1-R	CAGGCCATTGATCTCACGGT

Table S3 Comparison of six important amino acid residues between AtFT, AtTFL1 and OsMFT1

Position	AtFT	OsFT	AtTFL1	OsTFL1	OsMFT1	Potential function of OsMFT1
85	Y	Y	H	H	W	Partial FT activity
109	E	E	E	E	M	Strong TFL1 activity
128	L	L	K	K	K	Weak TFL1 activity
138	W	W	S	F/S	A	Strong TFL1 activity
140	Q	Q	D	D	A	No TFL1 activity
152	N	N	D	D/E	D	No TFL1 activity

Amino acid positions indicated are based on the Arabidopsis FT protein sequence. OsFT indicates Hd3a and RFT1, OsTFL1 indicates RCN1, RCN2, RCN3 and RCN4. As descripted in previous study, the effects of single amino acid residues substitution of AtFT are as follows (Ho and Weigel, 2014). Y85H had a weak TFL1-like activity and Y85W had partial FT activity; E109M caused FT to behave like TFL1; L128K bestowed weak TFL1-like activity on the mutant protein; W138S and W138A conferred repressive activity on AtFT; Q140D and Q140A are negative and neutral substitutions; N152D is negative substitutions.
